# Cardiac Resynchronization Therapy and Conduction System Pacing

**DOI:** 10.3390/jcm14093212

**Published:** 2025-05-06

**Authors:** Thomas Garvey O’Neill, Takahiro Tsushima, Bhupendar Tayal

**Affiliations:** 1 Department of Medicine, University Hospitals Cleveland Medical Center, Case Western Reserve University School of Medicine, Cleveland, OH 44106, USA; 2 Department of Medicine, Division of Cardiology, University Hospitals Harrington Heart & Vascular Institute, Case Western Reserve University School of Medicine, Cleveland, OH 44106, USA; 3 Department of Medicine, Division of Cardiology, University of Arkansas Medical Center, Little Rock, AR 72205, USA

**Keywords:** conduction system pacing, cardiac resynchronization therapy, left bundle branch block

## Abstract

Left bundle branch block (LBBB), initially described in the early 20th century, has become increasingly recognized as one of the leading causes of advanced heart failure (HF). In addition to rapidly growing data on guideline-directed medical therapy, cardiac resynchronization therapy (CRT) via transvenous coronary sinus lead has been the gold-standard therapy, but one-third of the indicated patients do not receive the expected benefits. Recently, cardiac conduction system pacing (CSP) was identified as an alternative to traditional CRT strategy, and multiple data have been published during the last few years. This review will discuss the diagnostic criteria of LBBB and its relation to the development of HF and review available data for traditional CRT as well as CSP in depth.

## 1. Introduction

Normal cardiac conduction rapidly activates both ventricles via the bundle of His (penetrating portion of the AV bundle) and subendocardial Purkinje fibers of the right and left bundle branches [1]. A structural and/or functional block below the bundle of His, specifically on the left side, is known as left bundle branch block (LBBB). Chronic LBBB causes pathological remodeling in patients with advanced cardiomyopathy and is significantly associated with adverse outcomes [2,3,4]. When the LBBB is refractory to guideline-directed medical therapy, the mainstream therapy is to restore intracardiac activation with a cardiac implantable electronic device (CIED), a strategy known as cardiac resynchronization therapy (CRT) or biventricular synchronization. Traditional CRT uses a transvenous coronary sinus (CS) lead, but newer techniques attempt more physiologic cardiac activation and are generally known as cardiac conduction system pacing (CSP). Herein, we will discuss the history, available data, known advantages, and potential dis-advantages of each therapy in detail.

## 2. Pathophysiology of Left Bundle Branch Block and Heart Failure

An intact cardiac conduction system is characterized by rapid propagation of electrical signals, due to the relatively high density of gap junction proteins, specifically connexin 43, through cells optimized for faster transmission of signals in the Purkinje system [5]. In a normal substrate, electrical signals from the His bundle are preferentially conducted to the left bundle branch (LBB) initially due to its shorter refractory period in comparison to the right bundle branch (RBB) at rest. The electrical propagations produce left-to-right activation of the basal interventricular septum (IVS) followed by mechanical contractions of both ventricles [1]. These electro-mechanical activations manifest as an initial contraction of the left ventricle (LV) followed by the slightly delayed right ventricle (RV) contraction realigning the LV and the outflow tract.

Contrarily, the electrical activation in patients with LBBB starts from the RV endocardium and reaches the LV endocardium through the IVS by myocyte-to-myocyte propagation of the electrical impulse without utilizing the cardiac conduction system [1]. Because such intracardiac conductions depend on ventricular myocytes (cell-to-cell propagation) predominantly, they lead to significant inter/intraventricular dyssynchrony [1]. Those unbalanced contractions of the RV and IVS during the early stages of ventricular systole lead to the leftward displacement of the IVS and the forceful displacement of blood towards the lateral wall [6]. The contraction of the IVS without the opposing forces of the LV leads to premature closing of the mitral valve, without the normal pressure rise. The displacement of blood towards the lateral wall causes an exaggerated stretch response and a powerful contraction, which bows the IVS back towards the RV. The increased stretch and compensatory contraction ultimately lead to hypertrophy of the LV lateral wall [7]. This inefficient activation of the LV with an unproductive myocardial distribution eventually results in the development of HF in LBBB causing ventricular dysfunction, discoordination, and remodeling [8]. Over time, the relative difference in myocardial work between the IVS and lateral wall leads to differences in blood flow, in which the septum receives reduced perfusion relative to the lateral wall in LBBB [4].

**Figure 1:**
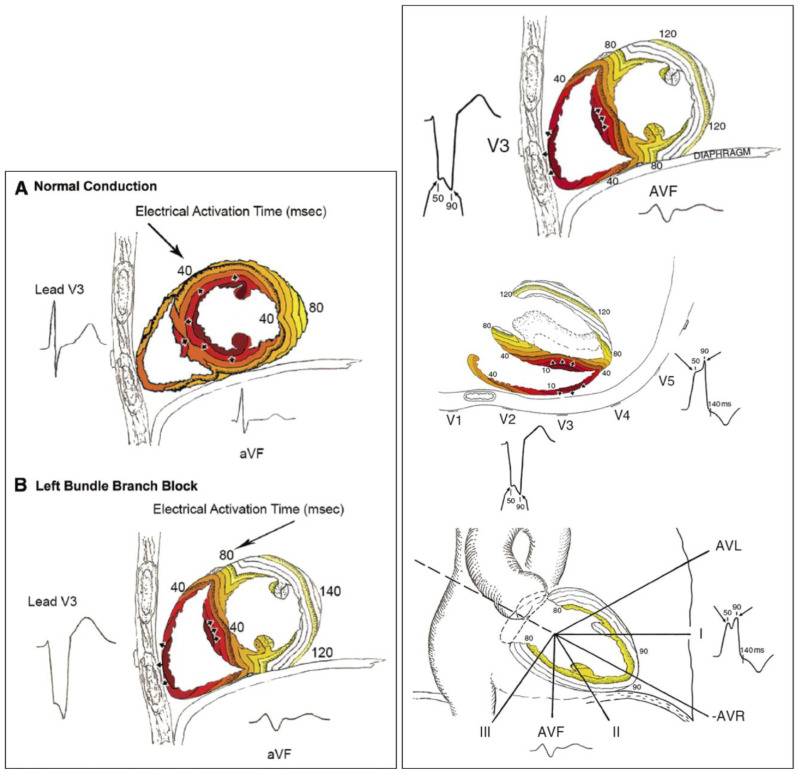
Demonstration of conduction patterns in normal (**A**) and delayed (**B**) conduction systems, as well as the subsequent ECG findings in lead V3 and aVF. Reprinted with permission from Ref. [1] 2011, Strauss.

These pathological changes with LBBB manifest as a distinctive pattern on an ECG, with a wide QRS complex. The prolonged QRS width with LBBB corresponds to the delayed depolarization of the LV. As highlighted above, it is estimated that the incidence of LBBB in patients with HF is greater than 30% [7]. As Strauss et al. outlined in their 2011 review, normal conduction begins in the LV endocardium and completes biventricular activation within approximately 80 ms [1]. The activation of LBBB begins in the RV endocardium and takes approximately >140 ms to complete the activation of the whole LV [1] (Figure 1).

## 3. Left Bundle Branch: Electrocardiographic Criteria

Although LBBB was identified more than 100 years ago, the precise criteria continued to be fine-tuned over the subsequent century. The American Heart Association (AHA), the American College of Cardiology (ACC), and the Heart Rhythm Society (HRS) provided a definition in 2009, updated most recently in 2018, which is outlined below (Table 1) [9]. To improve the diagnostic specificity, Strauss et al. proposed an alternative set of parameters to identify patients who would be more likely to respond to CRT [1]. Their definition of LBBB, included in full in Table 1 below, is notable for several differences from the AHA/ACC/HRS criteria. One significant recommendation is their advocating for a longer QRS, of ≥130 ms in women and 140 ms in men [1]. Other groups have attempted to further clarify a definition. Notably, in 2024, Treger et al. found that a time to notch in lead I greater than 75 ms was associated with improved specificity when compared with conventional criteria [10]. Strauss et al. note that nearly one-third of patients identified with LBBB based on traditional ECG criteria did not have electro-mechanical activation consistent with LBBB when evaluated with endocardial mapping [11]. These patients often had some degree of concurrent LV hypertrophy, non-specific intraventricular conduction delay, and/or pre-existing myocardial scarring [12]. Thus, the conventional criteria of electrocardiographic LBBB do not have enough accuracy to identify patients with true LBBB.

## 4. Non-Electrocardiographic Assessment for Left Bundle Branch Block

In the era of device therapies, it is important to know whether HF is caused by the LBBB or if the LBBB is merely a bystander. The above-described stricter ECG criteria may help to determine some of this, but non-invasive cardiac imaging can further help in ascertaining it [11]. HF patients often demonstrate dyssynchrony due to myocardial fibrosis or poor perfusion of the myocardium [12]. Utilization of imaging modalities may assist us in discerning whether the LBBB identified on the EKG is causing HF. The initial imaging-based methods focused primarily on identifying dyssynchrony in general by utilizing various echocardiographic techniques including M-mode, spectral Doppler, tissue Doppler imaging, and two- and three-dimensional speckle tracking echocardiography; they each rely on time-to-peak differences between the LV’s opposing walls to measure dyssynchrony [11,13]. All these methods had substantial inter-reader variability and failed to demonstrate any real-world application [14]. Moreover, these methods did not focus on identifying a true substrate for cardiac devices. The true role of cardiac imaging is to assist in clarifying whether LBBB is causing heart failure, and this can be done by directing attention towards the pathophysiological activation pattern noted in patients with LBBB as described above which finally results in a decline in LV systolic function and HF. Recently, some imaging-based methods like ‘true LBBB contraction pattern’ [15] or ‘septal flash/apical rocking’ [16] have shown some promising results that can provide aid beyond EKG in patient selection. However, the effectiveness of these methods has not yet been evaluated in randomized settings [17].

## 5. Biventricular Resynchronization Therapy with Traditional CRT via Transvenous CS Lead (BiV-CRT)

As the previous sections summarized in detail, abnormal intracardiac conduction such as chronic LBBB or high RV pacing by a cardiac implantable electronic device (CIED) tends to result in ventricular dyssynchrony or abnormal-conduction-induced cardiomyopathy. The mainstream therapy is to restore normal intracardiac conduction by the CIED, and the strategy has been called cardiac resynchronization therapy (CRT) or biventricular synchronization. For this purpose, currently, there are four available device treatments: biventricular synchronization via a transvenous coronary sinus (CS) lead (traditional CRT or BiV-CRT) as well as conduction system pacing (CSP) including His-bundle pacing (HBP), left bundle area pacing (LBAP), and a combination of these treatments (HBP and CS lead [HOT-CRT] or LBAP and CS lead [LOT-CRT]).

The traditional CRT (BiV-CRT) via a transvenous CS lead is aimed at depolarizing the left ventricle (LV) basal lateral or postero-lateral wall epicardially earlier than the intrinsic LV activation. As the previous section emphasized, the LV basal lateral or postero-lateral myocardium had the latest activation in the setting of chronic LBBB. Either the patient’s intrinsic His–Purkinje antegrade conduction or RV apical pacing was fused with the LV pacing to generate the biventricular propagation. The first BiV-CRT demonstrated the benefits of using an epicardial LV lead in 1994 and the CS lead has been placed trans-venously since 1998 [7,18]. BiV-CRT has been supported by numerous amounts of observational studies and medium–large-scale landmark trials (Table 2).

## 6. Guideline Recommendations for Traditional or BiV-CRT Implantation

Based on these results, the most recent guidelines had class I recommendation of BiV-CRT in patients with symptomatic cardiomyopathy (left ventricular ejection fraction (LVEF) < 35%) refractory to GDMT, New York Heart Association (NYHA) functional status II up to ambulatory IV status, and complete LBBB > 150 ms (Table 3) [28,29,30,31]. Importantly, in patients with incomplete LBBB (narrow QRS width: <120 ms), BiV-CRT showed higher mortality and adverse outcomes with or without echocardiographic assessment on mechanical LV dyssynchrony. Therefore, these patients should not undergo CRT implantation [28,29,30,31,32,33,34].

Because of the absence of head-to-head RCTs between CRT-D and CRT-P, the mortality benefits with either CIED subtype are still unclear. Per ESC Guidelines on cardiac pacing and CRT in 2021, the addition of an ICD lead to CRT can be considered, especially for patients with good survival prognosis due to a young age and less comorbidity, ischemic cardiomyopathy, and myocardial fibrosis on CMR [29]. Perhaps, CRT-P may be preferred for patients with non-ischemic cardiomyopathy, elder age and shorter life expectancy, multiple major comorbidities, and less myocardial fibrosis on CMR.

In advanced HF patients with non-LBBB such as RBBB or non-specific intraventricular conduction delay (IVCD), these populations were underrepresented in previous trials of BiV-CRT, and the conventional guideline suggested BiV-CRT implantation with class IIa-IIb recommendations based on a meta-analysis [23,28,29,30,31,35,36]. Of note, a recent post hoc analysis reported potential proarrhythmic effects of BiV-CRT, especially in patients with non-LBBB patients based on five landmark primary prevention ICD trials with a total of 2862 patients [37]. A potential explanation for the proarrhythmic effects of BiV-CRT in patients with non-LBBB was that BiV-CRT did not achieve greater reversed LV remodeling and led to the development of a proarrhythmic substrate secondary to increasing transmural dispersion of repolarization with epicardial pacing in comparison to patients with LBBB [35]. For these reasons, the decision for BiV-CRT implantation in HF patients with non-LBBB should be made carefully.

**Figure 2:**
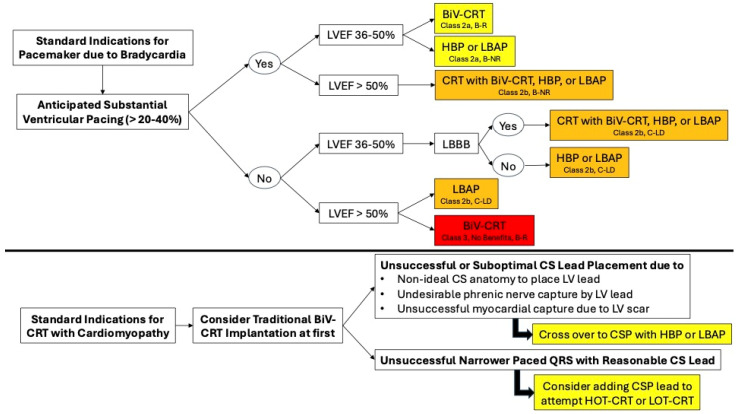
Central illustration: summary of optimal device therapy given clinical presentation. BiV-CRT = traditional CRT; CS = coronary sinus; CSP = conduction system pacing; HBP = His-bundle pacing; LBAP = left bundle area pacing; LV = left ventricle; LVEF = left ventricular ejection fraction.

**Figure 3:**
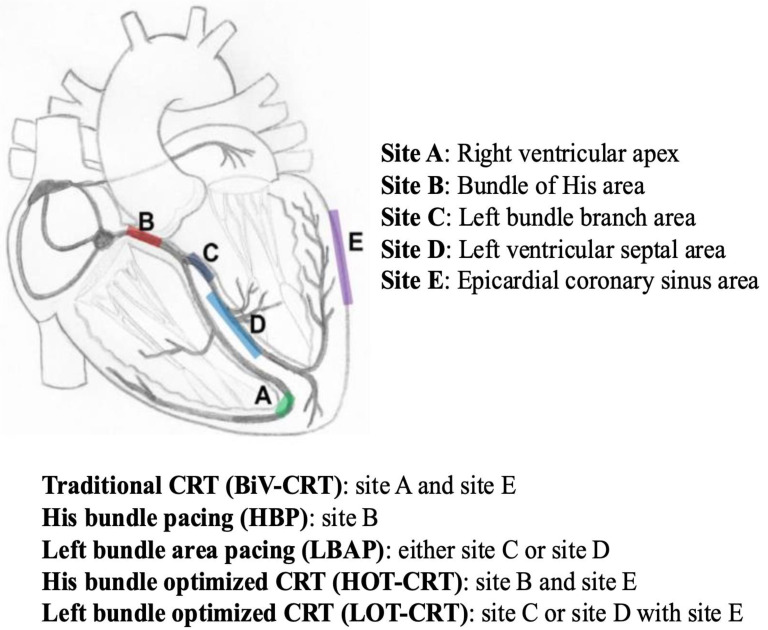
Demonstration of the ideal lead placement for traditional CRT, as well as the various conduction system pacing options His-bundle pacing (HBP), left bundle area pacing (LBAP), His-bundle optimized CRT (HOT-CRT), and left bundle optimized CRT (LOT-CRT). This figure was modified from Nguyen et al., 2024 [38].

In patients with an LVEF less than 50% with an expected high RV pacing burden (>20–40%), a BiV-CRT implantation or upgrade from the previously implanted CIED system was highly recommended (Figure 2). The lead placement in the different device therapies is summarized in the figure below (Figure 3). For a more detailed decision-making algorithm, readers are encouraged to consider the figures contained within the 2023 Heart Rhythm Society Guideline on Cardiac Physiologic Pacing for the Avoidance and Mitigation of Heart Failure [31].

## 7. Suggested Mechanism of Traditional BiV-CRT Non-Responders

**Figure 4:**
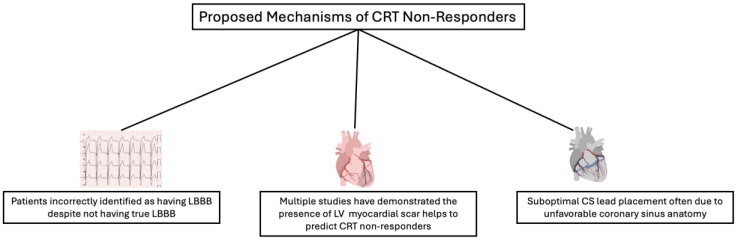
Depiction of the proposed mechanisms for CRT non-responders.

Approximately one-third of traditional CRT receivers did not demonstrate the expected improvements in clinical HF symptoms and/or echocardiographic LV reversed remodeling [39,40]. The mechanism of CRT non-responders will be multifactorial, as summarized below (Figure 4).

1. Lack of true substrate for CRT

CRT involves a device that mechanically corrects the problems caused by the non-functional physiological left bundle with an additional lead. As the previous sections explained in detail, QRS width and typical ECG morphology such as Strauss criteria for LBBB had been used as a surrogate for LV mechanical dyssynchrony. As explained earlier, the surface ECG can sometimes be misleading, and there may not be true LBBB. Secondly, even if LBBB is truly present, it may be just a bystander and may not be causing the HF. Therefore, a true substrate for CRT represents a case of LBBB cardiomyopathy that is very difficult to establish.

2. Presence and size of LV myocardial scarring

The MADIT-CRT trial sub-study reported that the absence of prior myocardial infarction highly predicted CRT super-responders (LVEF change > 14.5%) [41]. Tao et al. reported that a non-U-shaped LV contraction pattern correlated with the presence of myocardial scarring and the lack of CRT response using gated single-photon-emission computed tomography myocardial perfusion imaging [42]. Cardiac magnetic resonance imaging also quantified the amount of myocardial scarring associated with a poor response to CRT [43]. However, a cutoff of scar burden predicting CRT non-responders has not been validated [39,43].

3. CS lead position

Suboptimal CS lead placement is also related to CRT non-responders. The placement of a transvenous CS lead has to overcome multiple difficulties with 1. finding or passing the CS ostium due to a prominent Eustachian or web-like Thebesian valve; 2. poor target veins for the CS lead placement; 3. a high pacing threshold due to an adjacent LV scarring area; and/or 4. undesirable phrenic nerve capture in an otherwise suitable position [44].

In summary, the evidence for the traditional Biv-CRT is well supported by large randomized clinical trials, but clinical outcomes of Biv-CRT can be negatively affected by non-reversible cardiac problems such as a lack of true substrate, LV scarring, and a suboptimal CS lead position, as summarized above. For these reasons, clinical interests shifted to CSP for the last decade.

## 8. His-Bundle Pacing (HBP)

It had been well known that His–Purkinje activation can provide synchronous biventricular contraction and preserve LV systolic function based on animal models in comparison to non-physiologic asynchronous RV apical pacing. Therefore, HBP was originally intended to prevent high-RV-pacing-related cardiomyopathy. In 2000, the first HBP in humans was performed for eighteen patients with dilated cardiomyopathy who required AV nodal ablation for permanent atrial fibrillation (AF) with subsequent permanent pacemaker implantation [45]. A recent meta-analysis reported that HBP demonstrated improvements in LVEF, a narrower paced QRS width, and a low risk of HF hospitalization in comparison to traditional RV pacing [46]. Because almost one-third of traditional BiV-CRT-indicated patients resulted in CRT non-responders, HBP was investigated as an alternative to BiV-CRT in a relatively small number of predominantly observational studies, as summarized below. In general, HBP and HOT-CRT pacing systems demonstrated better outcomes in comparison to traditional BiV-CRT.

However, HBP has several disadvantages such as 1. a higher risk for macro- or micro-lead dislodgements, 2. a need for lead revisions (5–7%) due to inappropriate sensing of right atrial signals instead of true His potential, 3. a chronologically increased His pacing threshold and shortened battery longevity, and 4. a relatively lower chance of successful lead implantation (less than 90%) [47]. Even though HBP truly captured the His–Purkinje conduction system, more than 20% of patients did not achieve a narrower paced QRS width due to the concurrent intra-/infra-His conduction disease [47]. These procedural difficulties are some of the reasons why LBAP was quickly adapted as an alternative to BiV-CRT recently (Table 4).

## 9. Left Bundle Area Pacing (LBAP)

LBAP was first described in 2017 as an alternative to HBP [56]. Because of the shorter procedural time and stable lead functions, this technique was quickly adopted by experienced electrophysiologists. As summarized in the table below, the LBAP demonstrated 1. better electrical and mechanical synchrony of the ventricles, 2. low and stable pacing thresholds, 3. the potential to treat more distal conduction system disease, 4. reductions in all-cause mortality and HF-related hospital admissions, and 5. possibly a faster learning curve in comparison to HSP. Contrary to HBP or HOT-CRT pacing systems, the currently available data for LBAP are supported by a much larger number of observational studies, but large-size clinical trials are limited. Based on these data, the LBAP system has been implanted in patients with standard indications for traditional BiV-CRT implantation and/or a history of failed BiV-CRT due to procedural difficulties or poor response to the previously implanted CS lead pacing (Table 5).

## 10. Ongoing Randomized Clinical Trials for Conduction System Pacing

There are multiple ongoing randomized trials examining the role of CSP covering most of the clinical situations, and the basic information of each trial is summarized below. These studies will help establish the future practice for CSP for the following patients:

- Patients requiring pacing treatment for high-degree AVB (Table 6);
- Patients requiring CRT therapy following the standard indications (Table 7);
- Patients with permanent AF with slow ventricular response and/or requiring AV nodal ablation for better rate control (Table 8);
- Patients with acute post-procedural AVB secondary to transcatheter aortic valve implantation (Table 9).

## 11. Uncertainty with CSP and Interests in Future Investigations

Because the use of a conventional CSP system is a relatively new technique, there are several uncertainties, and further investigations are required. A recent meta-analysis published in 2023 evaluated 21 studies, including four randomized control trials. Although the randomized trials included in the analysis were relatively small, with the number of subjects ranging from 40 to 70, the meta-analysis demonstrated a significant reduction in all-cause mortality (odds ratio of 0.68, 95% confidence interval [CI] of 0.56–0.83), as well as a reduction in heart failure hospitalizations (odds ratio 0.52, 95% CI: 0.44–0.63) [69]. This analysis suggests the possibility of improved outcomes with CSP when compared with traditional BiV-CRT; however, further, larger randomized trials are still needed to further clarify these findings.

There remain several questions that will require continued interrogations. Some of these are highlighted as follows, but they include differences in clinical outcomes between the different modalities of CSP, technical and procedural differences within modalities, and CSP effects for patients with heart failure with preserved ejection fraction:

- Optimal criteria for determining a successfully captured cardiac conduction system during the CSP implantation, especially for the LBAP system;
- Differences in clinical outcomes between proximal and distal LBAP;
- Differences in clinical outcomes between LBAP and LV septal pacing;
- Delayed activation of the right ventricle with LBAP, and whether anodal capture of the LBAP lead is good or bad in patients with concurrent RV failure;
- Benefits of CSP in patients with diastolic heart failure;
- Safety and long-term outcomes of the CSP implantation in patients with interventricular septal disease such as infiltrative cardiomyopathy like cardiac sarcoidosis;
- Safety and short/long-term outcomes of transvenous lead extraction of CSP leads.

## 12. Conclusions

LBBB is the most common conduction disease in patients with advanced HF. Due to the abnormal electro-mechanical correlations, this acquired infra-Hisian conduction disease is associated with major cardiac adverse events. The standard treatment for LBBB refractory to GDMT was BiV-CRT, but its clinical effectiveness was not enough due to its procedural difficulties with the currently available equipment. In the last several years, LBAP with or without the traditional CS lead was rapidly adopted as an alternative CRT. Robust data from ongoing trials will provide new insights for CIED utilization in patients with advanced cardiomyopathy and cardiac conduction system disease in the future.

## Figures and Tables

**Table 1:** Conventional electrocardiographic criteria for left bundle branch block; AHA = American Heart Association; ACC = American College of Cardiology; HRS = Heart Rhythm Society.

ECG Findings	AHA/ACC/HRS Definition	Strauss et al. [1]
QRS duration	Complete: ≥120 ms Incomplete: <120 ms	≥140 ms (men), ≥130 ms (women)
Lateral leads (I, aVL, V5, V6)	Broad notched or slurred R waves Absent Q waves Occasional RS pattern in V5 and V6	Broad notched or slurred R waves
Precordial leads (V1, V2, V3)	Small initial r waves in V1, V2, and V3	Broad notched or slurred mid-QRS QS or rS in leads V1 and V2
R peak time in lateral leads	>60 ms in V5 and V6	Not applicable
ST and T waves	Usually opposite in direction to QRS Positive concordance	Not applicable

**Table 2:** Clinical data for BiV-CRT.

Patients with Cardiomyopathy					
Study Author	Study Design	Size	Population	Primary Endpoints	Outcomes
Abraham et al. NEJM 2002 [19]	Multicenter Prospective RCT	453 GDMT: 225 CRT:228	ICM or NICM LVEF < 35% QRS > 130 ms	NYHA status QOL score 6MWT	BiV-CRT improved patient functional status and ΔLVEF significantly.
Bristow et al. NEJM 2004 [20]	Multicenter Prospective RCT	1520 GDMT: 308 CRT-P: 617 CRT-D: 595	ICM or NICM LVEF < 35% NYHA III or IV QRS > 120 ms	Composite of - Overall death - Admission for any cause	BiV-CRT had 20% reduction in the primary endpoint.
Cleland et al. NEJM 2005 [18]	Multicenter Prospective RCT	813 GDMT: 404 CRT: 409	ICM or NICM LVEF < 35% NYHA III or IV QRS > 120 ms	Composite of - Overall death - Admission for MACE	BiV-CRT had 37% reduction in the primary endpoint.
Linde et al. JACC 2008 [21]	Multicenter Prospective RCT	610 CRT-ON: 419 CRT-OFF: 191	ICM or NICM LVEF < 40% NYHA I or II QRS > 120 ms	HF clinical composite response	BiV-CRT improved the primary endpoint.
Moss et al. NEJM 2009 [22]	Multicenter Prospective RCT	1820 CRT-D: 1089 ICD: 731	ICM or NICM LVEF < 30% NYHA I or II QRS > 130 ms	Composite of - Overall death - Nonfatal HF event	CRT-D had 34% reduction in the primary endpoint.
Tang et al. NEJM 2010 [23]	Multicenter Prospective RCT	1798 ICD: 904 CRT-D: 894	ICM or NICM LVEF < 30% NYHA II or III QRS > 120 ms	Composite of - Overall death - Admission for HF	CRT-D had 25% reduction in the primary endpoint.
Patients with Standard Bradycardia Pacing Indication and Preserved LVEF					
Study Name	Study Design	Size	Population	Primary Endpoints	Outcomes
Yu et al. Eur J Heart Fail. 2009 [24]	Multicenter Prospective RCT	177 RV apex: 88 CRT: 89	LVEF > 45% Pacing for AVB	12-month changes in - LVESV - LVEF	BiV-CRT improved LVEF and decreased LVESV.
Stockburger et al. Eur J Heart Fail. 2011 [25]	Multicenter Prospective RCT	108 RV apex: 58 CRT: 50	Pacing for AVB Expected RV pacing burden > 80%	12-month change in LVEDV	BiV-CRT did not show improvements in the primary endpoint.
Curtis et al. NEJM 2013 [26]	Multicenter Prospective RCT	691 RV apex: 342 CRT: 349	LVEF < 50% NYHA I, II, III Pacing for AVB	Composite of - Overall death - HF-related admission - Increase in LVESV index	BiV-CRT had 26% reduction in the primary endpoint.
Yu et al. Eur J Heart Fail. 2014 [27]	Multicenter Prospective RCT	177 RV apex: 88 CRT: 89	LVEF > 45%	LVESV LVEF	BiV-CRT improved LVEF and decreased LVESV.

AVB = atrioventricular block; CRT = cardiac resynchronization therapy; CRT-D = CRT with defibrillator; CRT-P = CRT with pacemaker; GDMT = guideline-directed medical therapy; HF = heart failure; ICD = implantable cardioverter–defibrillator; ICM = ischemic cardiomyopathy; NICM = non-ischemic cardiomyopathy; LVEDV = left ventricular end-diastolic volume; LVEF = left ventricular ejection fraction; LVESV = left ventricular end-systolic volume; QOL = quality of life; RCT = randomized clinical trial; RV = right ventricle.

**Table 3:** Guideline recommendations for traditional BiV-CRT.

Rhythm	QRS Morphology	QRS Width	NYHA Functional Status	Level of Recommendation
Sinus	LBBB	>150 ms	II, III, Ambulatory IV	Class I
		>150 ms	I, LVEF < 30%, ICM	Class IIb
		120–149 ms	II, III, Ambulatory IV	Class IIa
		120–149 ms	I	Class III
		<120 ms	I, II, III, Ambulatory IV	Class III
	Non-LBBB	>150 ms	III, Ambulatory IV	Class IIa
		>150 ms	II	Class IIb
		>150 ms	I	Class III
		120–149 ms	III, Ambulatory IV	Class IIb
		120–149 ms	I, II	Class III
Atrial Fibrillation with Any QRS		>120 ms	III, Ambulatory IV	Class IIa
High RV Pacing Burden (>40%)		Any	I, II, III, Ambulatory IV	Class IIa

**Table 4:** Clinical data for HBP.

HBP vs. RV Apical Pacing					
Study Author	Study Design	Size	Major Findings [95% Confidence Interval]		
Sun et al. JCE 2020 [46]	Systematic Review Meta-Analysis	2348 cases 13 studies	Improvement of LVEF (MD: +5.65 [4.38–6.92]) Narrower paced QRS (MD: −39.29 [−41.90–−36.68]) Lower risk of HF-related admission (OR: 0.65 [0.44–0.96]) Higher pacing threshold (MD: +0.8 [0.71–0.89]) Unchanged LVEDV (MD: −0.05 [−6.71–6.60]) Unchanged LVESV (MD: −1.37 [−5.75–3.01])		
HBP vs. Traditional BiV CRT					
Study Author	Study Design	Size	Population	Primary Endpoints	Outcomes
Lustgarten et al. Heart Rhythm 2015 [48]	Multicenter Prospective Crossover Randomized	29 Both HBP and CS leads were implanted.	Standard indications for CRT	Feasibility Paced QRS width Quality of life NYHA status 6 min walk test LVEF	Primary endpoints were significantly improved by both HBP and Biv-CRT.
Upadhyay et al. JACC 2019 [49]	Multicenter Prospective RCT	41 HBP: 21 BiV-CRT: 20	Standard indications for CRT	Feasibility Paced QRS width ΔLVEF at 6 months CV death or admissions at 12 months	No significant differences between 2 groups
Vinther et al. JACC EP 2021 [50]	Single-Center Prospective RCT	50 HBP: 25 BiV-CRT: 25	Standard indications for CRT with LBBB	Successful HBP implantation	Successful implantation: HBP 72% vs. BiV-CRT: 96%
Huang et al. Heart Rhythm 2022 [51]	Multicenter Prospective RCT Crossover	50: All with both HBP and BiV-CRT	Persistent AF and reduced LVEF (<40%) undergoing AVN ablation	Change in LVEF	Significant ΔLVEF (HBP: 21.3% vs. BiV-CRT: 16.7%)
HOT-CRT (His-Bundle Optimized CRT) vs. Traditional BiV CRT					
Study Author	Study Design	Size	Population	Primary Endpoints	Outcomes
Vijayaraman et al. Circ EP 2019 [52]	Multicenter Observational Retrospective	27 HOT-CRT: 27	CLBBB (n = 17) IVCD (n = 5) with QRS >140 ms RV pacing (n = 5)	QRS width Echocardiographic parameter NYHA status	HOT-CRT showed - narrower paced QRS - better LVEF and NYHA status
Zweerink et al. JACC EP 2021 [53]	Single-Center Observational Prospective	19 HOT-CRT: 14 BiV CRT: 5	Standard indications for CRT	Reduction in LVAT	ΔLVAT Change: HOT-CRT: 66 ± 17 ms BiV-CRT: 90 ± 20 ms
Deshmukh et al. JCE 2020 [54]	Single-Center Observational Retrospective	21 HOT-CRT: 21	Standard indications for CRT HBP only not correcting the QRS		Paced QRS: HOT-CRT: 110 ± 14 ms BiV-CRT: 141 ± 15 ms
Vijayaraman et al. JACC EP 2023 [55]	Multicenter Prospective RCT	100 BiV-CRT: 50 HOT-CRT: 50	Standard indications for CRT LVEF < 50%	Change in LVEF at 6 months	ΔLVEF: HOT-CRT: 12.4 ±7.3% BiV-CRT: 8.0 ± 10.1% (*p* = 0.02)

AVB = atrioventricular block; AVN = atrioventricular node; CRT = cardiac resynchronization therapy; CRT-D = CRT with defibrillator; CRT-P = CRT with pacemaker; CV = cardiovascular; GDMT = guideline-directed medical therapy; HF = heart failure; ICD = implantable cardioverter–defibrillator; ICM = ischemic cardiomyopathy; NICM = non-ischemic cardiomyopathy; LVAT = left ventricular activation time; LVEDV = left ventricular end-diastolic volume; LVEF = left ventricular ejection fraction; LVESV = left ventricular end-systolic volume; MD = mean difference; QOL = quality of life; RCT = randomized clinical trial; RV = right ventricle.

**Table 5:** Clinical data for left bundle area pacing (LBAP).

LBAP vs. RV Apical Pacing					
Study Author	Study Design	Size	Major Findings [95% Confidence Interval]		
Sharma et al. Heart Rhythm 2022 [57]	Multicenter Observational Retrospective	LBAP: 321 RVP: 382	Composite outcome of overall death, HF admission, or upgrade to CRT: LBAP: 10.0% vs. RVP: 23.3% (HR: 0.46 [0.306–0.698]) Composite outcome, especially in patients with RV pacing > 20%: LBAP: 8.4% vs. RVP: 26.1% (HR: 0.32 [0.187–0.540])		
Leventopoulos et al. IJC 2023 [58]	Systematic Review Meta-Analysis	4250 25 studies LBAP: 2127 RVP: 2123	LBAP showed - HF admissions (OR: 0.33 [0.21–0.50]) - Overall death (OR: 0.52 [0.34–0.80]) - AF occurrence (OR: 0.43 [0.27–0.68])		
LBAP vs. Traditional BiV-CRT					
Study Author	Study Design	Size	Population	Primary Endpoints	Outcomes
Liang et al. Circ EP 2022 [59]	Multicenter Crossover Observational Prospective	21 LBAP: 21	ICM or NICM CLBBB Standard indications for CRT	Paced QRS width Paced QRS area Hemodynamic parameters	LBAP showed - greater reduction in paced QRS width and QRS area - hemodynamic improvements
“LEVEL-AT” Pujol-Lopez et al. JACC EP 2022 [60]	Multicenter Prospective RCT	70 CSP: 35 - HBP: 4 - LBAP: 31 BiV CRT: 35	Standard indications for CRT	Change in LVAT at 6-month follow-up	CSP (mainly LBAP) attained similar degrees of CRT.
Vijayaraman et al. JACC 2023 [61]	Multicenter Observational Retrospective	1778 Biv-CRT: 981 LBAP: 797	Standard indications for CRT	Composite of - Overall death - HF admission	LBAP had 26% reduction in the primary endpoint.
Diaz et al. JACC EP 2023 [62]	Multicenter Observational Prospective	371 BiV-CRT: 243 LBAP: 128	Standard indications for CRT	Composite of - Overall death - HF admission	LBAP had 38% reduction in the primary endpoint.
Diaz et al. JACC EP 2024 [63]	Multicenter Observational Prospective	415 BiV-CRT: 243 LBAP: 141 LVSP: 31	Standard indications for CRT	Composite of - Overall death - HF admission	LBAP reduced primary composite outcomes significantly. No significant differences in clinical outcomes between LVSP and BiV-CRT.
Vijayaraman et al. Heart Rhythm 2024 [64]	Multicenter Observational Retrospective	1004 BiV-CRT: 178 HBP: 154 LBAP: 672	Standard indications for CRT NYHA II-IV Baseline LVEF: 36–50%	Composite of - Death - HF admissions	CSP (predominantly LBAP) had 36% reduction in the primary composite endpoint.
LOT-CRT (Left Bundle Optimized CRT) vs. Traditional BiV CRT					
Study Author	Study Design	Size	Population	Primary Endpoints	Outcomes
Jastzebski et al. Heart Rhythm 2021 [65]	Multicenter Observational Prospective	112 LOT-CRT: 91	Standard indications for CRT BiV CRT non-responders		LOT-CRT was better than BiV-CRT for - narrower paced QRS - ΔLVEF > 3 month - improvement on NYHA status > 3 months
Feng et al. BMC Cardiovasc Disord 2022 [66]	Single-Center Observational Prospective	21 LOT-CRT: 10 BiV CRT: 11	Standard indications for CRT		LOT-CRT was better than BiV-CRT for - narrower paced QRS - ΔLVEF > 9 month
Wang et al. JACC 2022 [67]	Multicenter Prospective RCT	40 LOT-CRT: 20 BiV-CRT: 20	NICM NSR LBBB NYHA II-IV	LVEF at 6 months	Significant ΔLVEF at 6 months (MD: 5.6% [0.3–10.9])
Jastrzebski et al. Circ EP 2024 [68]	Crossover Multicenter Prospective Observational Open-Label	48	Standard indications for CRT Cardiomyopathy NSR IVCD: 29 LBBB: 19	LV dP/dt _max_ from baseline to BiV-CRT, LBAP, or LOT-CRT	In patients with advanced conduction diseases, both LOT-CRT and BVP showed greater hemodynamic benefits than LBAP alone.

AVB = atrioventricular block; AVN = atrioventricular node; CRT = cardiac resynchronization therapy; CRT-D = CRT with defibrillator; CRT-P = CRT with pacemaker; CV = cardiovascular; GDMT = guideline-directed medical therapy; HF = heart failure; ICD = implantable cardioverter–defibrillator; ICM = ischemic cardiomyopathy; IVCD = interventricular conduction delay; NICM = non-ischemic cardiomyopathy; LBBB = left bundle branch block; LVAT = left ventricular activation time; LV dP/dt_max_ = left ventricular pressure maximal first derivative; LVEDV = left ventricular end-diastolic volume; LVEF = left ventricular ejection fraction; LVESV = left ventricular end-systolic volume; LVSP = left ventricular septal pacing; MD = mean difference; NSR = normal sinus rhythm; QOL = quality of life; RCT = randomized clinical trial; RV = right ventricle.

**Table 6:** Ongoing or planned clinical trials for conduction system pacing for bradyarrhythmias.

Study	Study Design	Size	Population	Endpoints
NCT05015660 Canada Recruiting	Single-center Prospective RCT Single-blinded	1300 LBAP vs. RVP (1:1)	LVEF > 35% Standard indications for pacemakers Anticipated RV pacing >90%	Primary: at 36 months - Time to cardiovascular death - Time to HF admission - Worsening LVESV index Secondary: at 22 months - Overall and CV mortality - HF admissions - QOL improvement
Spain Recruiting	Single-center Prospective RCT Single-blinded	200 HBP/LBAP vs. RVP (1:1)	LVEF > 50% Standard indications for pacemakers	Primary: at 12 months - Incidence of pacemaker-induced cardiomyopathy Secondary: at 12 months - Change in LVESV - New HF admissions
“LEAP” NCT04595487 Europe Recruiting	Multicenter Prospective RCT Single-blinded	470 LVSP vs. RVP (1:1)	LVEF > 40% Standard indications for pacemakers Expected RV pacing > 20%	Primary: at 12 months - Composite of all-cause death, HF admissions, LVEF decline (> 10%) Secondary: at 12 months - New HF admissions - All-cause death - De novo AF - QOL analysis
“PROTECT-SYNC” NCT05585411 Korea Recruiting	Multicenter Prospective RCT Double-blinded	450 LBAP vs. RVP (1:1)	Standard indications for pacemakers Expected RV pacing > 40%	Primary: at 24 months - Composite of all-cause death, HF admissions, occurrence of pacing cardiomyopathy Secondary: at 24 months - New HF admissions - All-cause and CV death - De novo AF - QOL analysis
“LEAP-Block” NCT04730921 China Recruiting	Multicenter Prospective RCT Double-blinded	458 LBAP vs. RVP (1:1)	Standard indications for pacemakers Expected RV pacing > 40%	Primary: at 24 months - Composite of all-cause death, HF admissions, occurrence of pacing cardiomyopathy Secondary: at 24 months - Composite of new LVEF < 50% and/or increase in LVESV > 15% - QOL analysis
“OptimPacing” NCT04624763 China Recruiting	Multicenter Prospective RCT Double-blinded	683 LBAP vs. RVP (1:1)	Standard indications for pacemakers LVEF > 35% NYHA I-III status	Primary: at 36 months - Composite of all-cause death, HF admissions, occurrence of pacing cardiomyopathy Secondary: at 36 months - LVEF, LVESV, and LVEDV - NYHA status - QOL analysis
“PROTECT-HF” NCT05815745 UK, Slovenia Recruiting	Multicenter Prospective RCT Double-blinded	2600 HBP/LBAP vs. RVP (1:1)	Standard indications for pacemakers LVEF > 35%	Primary: at 78 months - All-cause death - HF admissions Secondary: at 78 months - Upgrade to BiV-CRT - NYHA status - QOL analysis

AVB = atrioventricular block; AVN = atrioventricular node; CRT = cardiac resynchronization therapy; CRT-D = CRT with defibrillator; CRT-P = CRT with pacemaker; CV = cardiovascular; GDMT = guideline-directed medical therapy; HF = heart failure; ICD = implantable cardioverter–defibrillator; ICM = ischemic cardiomyopathy; IVCD = interventricular conduction delay; NICM = non-ischemic cardiomyopathy; LBBB = left bundle branch block; LVAT = left ventricular activation time; LV dP/dt_max_ = left ventricular pressure maximal first derivative; LVEDV = left ventricular end-diastolic volume; LVEF = left ventricular ejection fraction; LVESV = left ventricular end-systolic volume; LVSP = left ventricular septal pacing; MD = mean difference; NSR = normal sinus rhythm; QOL = quality of life; RCT = randomized clinical trial; RV = right ventricle.

**Table 7:** Ongoing or planned clinical trials for conduction system pacing for cardiac resynchronization therapy.

Study Name	Study Design	Size	Population	Endpoints
“LIT-HF” NCT05572957 China Recruiting	Multicenter Prospective RCT No blinding	50 HBP/LBAP vs. GDMT	Symptomatic NICM with LVEF < 35% Complete LBBB NYHA II-III status Standard indications for pacemakers LVEF > 35%	Primary: at 6 months - Requirements of new ICD for SCD - New reduction in LVEF < 35% Secondary: at 18 months - LVEF, LVESV, and LVEDV - NYHA status - QOL analysis
“HIS-CRT” NCT05265520 USA Recruiting	Multicenter Prospective RCT Single-blinded	120 HOT-CRT vs. BiV-CRT	LVEF < 35% with RBBB QRS >150 ms LVEF <35% with RBBB QRS 120–149 ms	Primary: at 6 months - Change in LVEF Secondary: at 6 months - Paced QRS width - LVESV and LVEDV
“REINVENT” NCT05652218 USA Completed recruiting/not published yet	Multicenter Crossover Prospective RCT Single-arm Double-blinded	21 LOT-CRT or BiV-CRT	Strict LBBB NYHA I-IV status LVEF > 35% on stable GDMT	Primary: at 6 months - Change in myocardial performance index Secondary: - None
“HIS-alt_2” NCT04409119 Denmark Recruiting	Multicenter Prospective RCT Single-blinded	125 HBP/LBAP vs. BiV-CRT	NICM LVEF < 35% Typical LBBB NYHA II-IV status Planned CRT-P/CRT-D upgrade following standard indications	Primary: at 6 months - Change in LVESV - Successful rate of narrowed paced QRS width with HBP or LBAP Secondary: at 6 months - LVEF and LVEDD - NYHA status - QOL
“LBBAP-AFHF” NCT05549544 China Recruiting	Multicenter Prospective RCT Double-blinded	60 LBAP vs. BiV-CRT	LVEF <50% with 3-month GDMT Permanent AF with planned AVN ablation Permanent AF with slow ventricular rate (anticipated RV pacing > 40%)	Primary: at 6 months - Change in LVEF Secondary: at 6 months - ΔLVEDD and ΔLVEDV -LVEF increase > 5–15% - Composite of all-cause death and/or HF admissions
“CSP-SYNC” NCT05155865 Slovenia Completed recruiting completed/not published yet	Single-center Prospective RCT Open-label	62 LBAP vs. BiV-CRT	LVEF < 35% with 3-month GDMT CLBBB NYHA II-III status	Primary: at 12 months - Change in LV volume and LVEF - 6 min walk test Secondary: at 12 months - Myocardial work redistribution - Paced QRS width - Arrhythmia occurrence
“CONSYST-CRT” NCT05187611 Spain Completed recruiting/not published yet	Multicenter Prospective RCT Non-inferiority	130 HBP/LBAP vs. BiV-CRT	LVEF < 35% with 3-month GDMT LBBB QRS > 130 ms IVCD QRS > 150 ms NYHA III-IV status	Primary: at 12 months - Composite of all-cause death, heart transplant, HF admissions, and LVEF improvement (>5%) Secondary: at 12 months - LVEF and LVESV - Paced QRS width - NYHA status
“Safety and Effectiveness of Left Bundle Branch Pacing in Patients With Cardiac Dysfunction and AV Block” NCT05553626 China Not yet recruiting	Multicenter Open-label Prospective RCT	160 LBAP vs. BiV-CRT	LVEF < 50% NYHA I-III Second or third AVB Anticipated RV pacing > 40%	Primary: at 12 months - Change in LVEF Secondary: at 12 months - Change in LVESV - Implant success - All-cause death and/or HF admissions - Paced QRS width
“LeCaRT” NCT05365568 Belgium Active/not recruiting	Multicenter Open-label Prospective RCT	170 LBAP vs. BiV-CRT	Standard indications for CRT NYHA II-IV LBBB QRS > 130 ms IVCD QRS > 150 ms Wide paced QRS	Primary: at 12 months - Composite of all-cause death, HF admissions, implant failure, and CIED re-intervention Secondary: at 12 months - Procedure and fluoroscopy time - Paced QRS width - 6 min walk - ICD therapy
“LEFT-BUNDLE-CRT” NCT05434962 Spain Recruiting	Multicenter Non-inferiority Open-label Prospective RCT	176 LBAP/LOT-CRT vs. BiV-CRT	Standard indications for CRT (class I or IIa) LBBB	Primary: at 12 months - CRT response Secondary: at 12 months - LVEF - Clinical outcomes - Death, HF admissions, heart transplantation, or VT/VF events
“PhysioSync-HF” NCT05572736 Brazil Active/not recruiting	Multicenter Prospective RCT Double-blinded	304 HBP/LBAP vs. BiV-CRT	LVEF < 35% NYHA II-III status LBBB QRS > 130 ms Standard indications for CRT	Primary: at 12 months - Composite of all-cause death, HF admissions, and LVEF change Secondary: at 12 months - Cost-effectiveness - Composite of all-cause death, HF admissions, and urgent HF visit
“Left vs. Left Randomized Clinical Trial” NCT05650658 USA, Canada Recruiting	Multicenter Prospective RCT Triple-blinded	2136 HBP/LBAP vs. BiV-CRT	LVEF < 50% QRS > 130 ms Upgrade to CRT due to RV pacing > 40%	Primary: at 66 months - Composite of all-cause death and HF admissions Secondary: at 66 months - QOL - Clinical outcomes - Changes in LVESV index - NYHA status - Appropriate ICD therapy

AVB = atrioventricular block; AVN = atrioventricular node; CRT = cardiac resynchronization therapy; CRT-D = CRT with defibrillator; CRT-P = CRT with pacemaker; CV = cardiovascular; GDMT = guideline-directed medical therapy; HF = heart failure; ICD = implantable cardioverter–defibrillator; ICM = ischemic cardiomyopathy; IVCD = interventricular conduction delay; NICM = non-ischemic cardiomyopathy; LBBB = left bundle branch block; LVAT = left ventricular activation time; LV dP/dt_max_ = left ventricular pressure maximal first derivative; LVEDV = left ventricular end-diastolic volume; LVEF = left ventricular ejection fraction; LVESV = left ventricular end-systolic volume; LVSP = left ventricular septal pacing; MD = mean difference; NSR = normal sinus rhythm; QOL = quality of life; RCT = randomized clinical trial; RV = right ventricle.

**Table 8:** Ongoing or planned clinical trials for conduction system pacing for atrial fibrillation.

Study Name	Study Design	Size	Population	Endpoints
“LBBAP-AFHF” NCT05549544 China Recruiting	Multicenter Double-blinded Prospective RCT	60 LBAP vs. BiV-CRT	LVEF < 50% NYHA II-IV status Permanent AF QRS < 130 ms Anticipated RV pacing > 40% due to planned AVN ablation or slow ventricular rate	Primary: at 6 months - ΔLVEF Secondary: at 6 months - Implant success rate - ΔLVEDD and ΔLVEDV - All-cause death and HF admissions
“CONDUCT-AF” NCT05467163 Europe Recruiting	Multicenter Open-Label Prospective RCT	82 HBP/LBAP vs. BiV-CRT	LVEF < 50% QRS < 120 ms Permanent AF with anticipated AVN ablation after CIED implantation	Primary: at 24 months - ΔLVEF Secondary: at 24 months - HF admissions and/or cardiac death - ΔLVEDD and ΔLVEDV - NYHA status
“RAFT-P&A” NCT05428787 Canada Active/not recruiting	Multicenter Double-blinded Prospective RCT	284 LBAP vs. BiV-CRT AVN ablation after 4 weeks of CIED implantation	LVEF < 50% QRS < 120 ms NYHA I-Iva status Permanent AF considered for AVN ablation	Primary: at 12 months - ΔNT-proBNP Secondary: at 12 months - Composite of HF admissions and/or all-cause death - Change in QOL - ΔLVEF, ΔLVESV index, and ΔLV global longitudinal strain - NYHA status

AVB = atrioventricular block; AVN = atrioventricular node; CRT = cardiac resynchronization therapy; CRT-D = CRT with defibrillator; CRT-P = CRT with pacemaker; CV = cardiovascular; GDMT = guideline-directed medical therapy; HF = heart failure; ICD = implantable cardioverter–defibrillator; ICM = ischemic cardiomyopathy; IVCD = interventricular conduction delay; NICM = non-ischemic cardiomyopathy; LBBB = left bundle branch block; LVAT = left ventricular activation time; LV dP/dt_max_ = left ventricular pressure maximal first derivative; LVEDV = left ventricular end-diastolic volume; LVEF = left ventricular ejection fraction; LVESV = left ventricular end-systolic volume; LVSP = left ventricular septal pacing; MD = mean difference; NSR = normal sinus rhythm; QOL = quality of life; RCT = randomized clinical trial; RV = right ventricle.

**Table 9:** Ongoing or planned clinical trials for conduction system pacing after TAVI.

Study Name	Study Design	Size	Population	Endpoints
“PHYS-TAVI” NCT04482816 Spain Completed recruiting/not published yet	Single-center Double-blinded Prospective RCT	24 HBP/LBAP vs. RVP	Post-TAVI AVB LVEF > 50%	Primary: at 12 months - Composite of overall survival, improvement in NYHA status, and/or 6 min walk Secondary: at 12 months - ΔLVEF - Correction of echocardiographic asynchrony - HF admissions - Paced QRS width
“PLANET” NCT05024279 Germany Active/not recruiting	Single-center Double-blinded Prospective RCT	30 LBAP vs. RVP	Post-TAVI AVB Post-TAVI AF with anticipated RV pacing > 20% LVEF > 50%	Primary: at 24 months - Paced QRS width Secondary: at 24 months - Clinical outcomes - HF admissions - ΔLVEF and ΔLVEDD - NYHA status
“Left Bundle BRAVE” NCT05541679 USA Recruiting	Multicenter Crossover Double-blinded Prospective RCT	46 LBAP vs. RVP	Post-TAVI AVB LVEF > 50%	Primary: at 18 months - Change in LVEF and global longitudinal strain - Composite of LV septal or coronary perforation, lead dislodgement Secondary: at 18 months - Clinical outcomes - Echocardiographic parameters

AVB = atrioventricular block; AVN = atrioventricular node; CRT = cardiac resynchronization therapy; CRT-D = CRT with defibrillator; CRT-P = CRT with pacemaker; CV = cardiovascular; GDMT = guideline-directed medical therapy; HF = heart failure; ICD = implantable cardioverter–defibrillator; ICM = ischemic cardiomyopathy; IVCD = interventricular conduction delay; NICM = non-ischemic cardiomyopathy; LBBB = left bundle branch block; LVAT = left ventricular activation time; LV dP/dt_max_ = left ventricular pressure maximal first derivative; LVEDV = left ventricular end-diastolic volume; LVEF = left ventricular ejection fraction; LVESV = left ventricular end-systolic volume; LVSP = left ventricular septal pacing; MD = mean difference; NSR = normal sinus rhythm; QOL = quality of life; RCT = randomized clinical trial; RV = right ventricle.

## Data Availability

No new data were created or analyzed in this study. Data sharing is not applicable to this article.
